# Dose–response relationships between early postoperative nutrition and subsequent complications in gastrointestinal cancer: optimal intake ranges for energy and protein

**DOI:** 10.3389/fnut.2026.1826047

**Published:** 2026-05-13

**Authors:** Ziqi Liu, Yuntao Hao, Jialin Li, Min Zhao, Ji Zhang, Qian Lu, Yanfei Wang, Yanli Wang, Liqing Gong, Qi Yu, Zhaode Bu, Zhi Peng, Xiaojiang Wu, Yu Fang

**Affiliations:** 1 Key Laboratory of Carcinogenesis and Translational Research (Ministry of Education), Department of Clinical Nutrition, Peking University Cancer Hospital and Institute, Beijing, China; 2 Key Laboratory of Carcinogenesis and Translational Research (Ministry of Education), Gastrointestinal Cancer Center, Peking University Cancer Hospital and Institute, Beijing, China; 3 School of Nursing, Peking University, Beijing, China; 4 Key Laboratory of Carcinogenesis and Translational Research (Ministry of Education), Day Oncology Unit, Peking University Cancer Hospital and Institute, Beijing, China; 5 Key Laboratory of Carcinogenesis and Translational Research (Ministry of Education), Department of Gastrointestinal Oncology, Peking University Cancer Hospital and Institute, Beijing, China

**Keywords:** dose–response relationship, energy intake, gastrointestinal cancer surgery, postoperative nutrition, protein intake

## Abstract

**Background and aim:**

Postoperative complications remain common after major gastrointestinal (GI) cancer surgery. Although early nutritional support is a core component of enhanced recovery protocols, optimal postoperative targets for energy and protein intake remain uncertain. This study aimed to characterize dose–response relationships between early postoperative nutritional intake and postoperative complications.

**Methods:**

This prospective observational cohort included adults undergoing elective gastrectomy or partial colectomy for GI malignancy. Postoperative energy and protein intake were quantified during postoperative days 1-2 (POD 1-2). Overall postoperative complications occurring after POD 2 and within 30 days after surgery were the primary outcome; infectious complications were secondary. Nonlinear associations were examined using multivariable logistic regression with restricted cubic splines (RCS) as the primary method, supported by generalized additive and quadratic models. Analyses were adjusted for demographic, surgical, oncologic, and nutritional covariates, including GLIM-defined malnutrition. Optimal intake ranges were defined using a CI-overlap criterion with bootstrap validation.

**Results:**

Among 642 patients, early postoperative energy intake demonstrated a statistically significant nonlinear association with complication risk (*p*-for nonlinearity = 0.018). The nadir occurred at 14.4 kcal/kg/day, with an optimal intake range of 12.8–20.1 kcal/kg/day. Protein intake showed a less consistent association, without statistically significant evidence of nonlinearity (*p* = 0.166). The nadir occurred at 0.81 g/kg/day, with an optimal intake range of 0.55–1.51 g/kg/day, with no consistent upper harm threshold observed within routine clinical ranges.

**Conclusion:**

Early postoperative energy intake after GI cancer surgery demonstrated a robust nonlinear association with postoperative complications, with lowest predicted risk observed at moderate caloric provision. In contrast, protein intake showed a less consistent association and a wider estimated optimal range. These findings warrant further investigation of individualized, precision-oriented postoperative nutrition strategies in surgical oncology.

## 1 Introduction

Optimal perioperative nutritional support is a cornerstone of enhanced recovery after surgery (ERAS) programs and is closely associated with postoperative outcomes in patients undergoing gastrointestinal (GI) cancer surgery (1, 2). Malnutrition affects more than 40% of patients undergoing major GI surgery and is consistently associated with higher rates of postoperative complications, delayed functional recovery, prolonged hospitalization, and reduced long-term survival (1, 3, 4). In response, early postoperative feeding, encompassing both enteral and parenteral modalities during postoperative days 1-2 (POD 1-2), has become an integral component of modern surgical care (5–7). Despite widespread adoption of early nutritional support, precise quantitative targets for postoperative energy and protein delivery remain incompletely defined.

Current European Society for Clinical Nutrition and Metabolism (ESPEN) and American Society for Parenteral and Enteral Nutrition (ASPEN) guidelines reference weight-based estimates for energy and protein provision in acutely ill or postoperative patients, commonly approximating 25–30 kcal/kg/day for energy and 1.2–1.5 g/kg/day for protein when indirect calorimetry is unavailable, while emphasizing individualized adjustment according to metabolic status and clinical tolerance (8, 9). However, these recommendations are largely extrapolated from intensive care or heterogeneous medical populations rather than elective surgical cohorts, and they are generally based on categorical or threshold-based frameworks rather than continuous surgery-specific dose–response analyses (10–12). Consequently, whether such generalized targets are optimal during the immediate postoperative period after GI cancer surgery remains uncertain.

Both overfeeding and underfeeding may plausibly contribute to adverse outcomes. Excessive caloric delivery in the early postoperative or acute illness phase has been associated with hyperglycemia, fluid overload, hepatic steatosis, and increased metabolic stress, particularly among metabolically vulnerable patients such as those with malignancy (8, 13). Conversely, inadequate nutritional intake may exacerbate negative nitrogen balance, impair wound healing, and increase susceptibility to infectious complications and functional decline (8, 14). In patients with cancer, tumor- and inflammation-driven metabolic alterations may further narrow the therapeutic window for safe and effective nutrient provision (15, 16). Taken together, these considerations suggest that optimal postoperative intake may lie within a relatively constrained range, yet robust empirical evidence defining this range in surgical oncology populations is limited.

Evidences support that high-protein intake significantly reduce postoperative complications and length of hospital stay in patients with cancer (17). Observational studies in critically ill populations have also suggested potential linear benefits with increasing protein intake up to 2.0–2.5 g/kg/day (18, 19). However, precise quantitative targets for early postoperative protein intake, particularly during POD 1-2, remain poorly defined. Clarifying the dose–response relationship for protein in GI cancer surgery is therefore essential to optimize postoperative outcomes while avoiding potential adverse effects, including renal stress or metabolic burden (20, 21).

Prior studies have largely employed categorical or linear models, which may obscure non-linear dose–response patterns critical for defining optimal intake (22). Flexible modeling approaches, including restricted cubic splines (RCS) and generalized additive models (GAM), allow continuous characterization of exposure–response relationships without imposing rigid functional assumptions (23, 24). Despite the clinical importance of postoperative nutrition, few studies have systematically integrated these flexible modeling approaches to evaluate energy and protein targets specifically in patients undergoing GI cancer surgery.

In this context, we conducted a prospective observational cohort study of 642 patients undergoing major GI cancer surgery. Using flexible dose–response modeling approaches, we aimed to: (1) characterize the continuous relationships between early postoperative energy and protein intake (POD 1-2) and 30-day complication risk; (2) assess consistency across complementary nonlinear modeling strategies; and (3) estimate intake ranges associated with the lowest predicted complication risk. By integrating rigorous exposure measurement with advanced statistical modeling, this study seeks to provide surgery-specific evidence to inform refinement of early postoperative nutritional strategies in GI oncology patients.

## 2 Materials and methods

### 2.1 Study design and participants

This prospective observational cohort study enrolled consecutive adult patients undergoing elective GI cancer surgery at Peking University Cancer Hospital between January 2022 and June 2023. Eligible procedures included gastrectomy and partial colectomy for histologically confirmed malignancy. Patients were included if they received postoperative nutritional support and had complete nutritional intake records during POD 1-2. Exclusion criteria comprised missing outcome data, reoperation within the exposure window, or implausible nutritional intake values. Detailed inclusion and exclusion criteria are provided in Supplementary Table S1. The study protocol was approved by the Institutional Ethics Committee. All participants provided written informed consent. The study was conducted in accordance with the Declaration of Helsinki.

### 2.2 Nutritional assessment and exposure definitions

Postoperative nutritional intake was assessed during PODs 1-2, prior to the onset of postoperative complications. Total intake included oral, enteral, and parenteral nutrition, derived from standardized medical and nursing records. Primary exposures were defined as energy intake (kcal/kg/day) and protein intake (g/kg/day) using adjusted body weight.

### 2.3 Outcome definitions

The primary outcome was overall postoperative complications occurring after POD 2 and within 30 days after surgery. Complications diagnosed on POD 1-2 were excluded to ensure that exposure (nutritional intake during POD 1-2) preceded outcome occurrence. Infectious complications occurring after POD 2 served as the secondary outcome. All patients were followed for 30 days postoperatively, including after discharge. Complications were prospectively recorded and adjudicated using standardized GI surgery criteria. Anastomotic fistulas were classified as surgical complications; when accompanied by microbiologically or clinically confirmed infection, the infectious event was recorded separately.

Infectious complications were evaluated as a secondary endpoint and included surgical site infection, pneumonia, intra-abdominal abscess, sepsis, and central venous catheter-related infections. Infectious outcomes were analyzed as a secondary endpoint due to lower event frequency, with caution in interpretation for rare exposures.

### 2.4 Covariate assessment

Covariates were selected *a priori* based on clinical relevance and potential confounding of the association between early postoperative nutrition and complication risk. Models were adjusted for age, BMI, gender, cancer stage, surgery type, Global Leadership Initiative on Malnutrition (GLIM)-defined malnutrition, and preoperative neoadjuvant therapy. These variables reflect baseline demographic characteristics, nutritional status, disease severity, and operative complexity.

Additional variables were not included to avoid overadjustment and potential multicollinearity, particularly for biomarkers that may lie along the causal pathway between nutritional intake and postoperative outcomes.

### 2.5 Statistical analysis

#### 2.5.1 Primary dose–response analysis

Nonlinear associations between nutritional exposures and 30-day postoperative complication risk were evaluated using multivariable logistic regression with RCS as the primary modeling strategy. Three degrees of freedom were specified to allow flexible curve estimation while limiting overfitting given the number of outcome events. Likelihood ratio tests compared spline-based models with corresponding linear models to formally assess evidence of nonlinearity. All models were adjusted for the covariates described above.

#### 2.5.2 Secondary and sensitivity analyses

To assess robustness, two complementary modeling approaches were applied: GAMs; Thin-plate regression splines (basis dimension *k* = 4), with smoothing parameters estimated using restricted maximum likelihood. Quadratic regression models: Including both linear and squared exposure terms. Both methods were conducted as supportive sensitivity analyses of the primary RCS findings and were not intended for hypothesis testing.

#### 2.5.3 Estimation of nadir and optimal intake range

The nadir was defined as the exposure value associated with the minimum predicted complication risk derived from the RCS model. An optimal intake range was defined using a confidence-interval overlap criterion: exposure values for which the lower bound of the 95% confidence interval of predicted risk did not exceed the minimum predicted risk. This approach identifies intake levels statistically indistinguishable from the nadir. Bootstrap resampling (1,000 iterations) was performed to quantify uncertainty around nadir estimates and optimal intake ranges.

#### 2.5.4 Model diagnostics and validation

Model discrimination was evaluated using the area under the receiver operating characteristic curve (AUC). Multicollinearity was assessed using variance inflation factors (VIF), with values <5 considered acceptable.

#### 2.5.5 Secondary and subgroup analyses

Infectious complications were analyzed separately due to lower event frequency. Exploratory subgroup analyses were conducted according to GLIM-defined malnutrition, surgery type, and preoperative neoadjuvant therapy. These analyses were considered hypothesis-generating.

#### 2.5.6 Software

All analyses were conducted using R (version 4.4.0), employing the rms, mgcv., and boot packages for RCS modeling, GAM fitting and bootstrap resampling, respectively. Figures were generated using ggplot2 with consistent visualization of predicted risk curves, nadir estimates, and optimal intake ranges.

## 3 Results

### 3.1 Patient characteristics and clinical outcomes

A total of 642 patients were included in the analysis. Baseline demographic and clinical characteristics are summarized in Table 1. The mean age was 60.34 ± 10.99 years, and 220 patients (34.3%) were female. According to BMI classification, 53.0% had normal weight, 32.8% were pre-obese, and 11.7% were obese. Advanced cancer stage (III-IV) was present in 49.9% of patients. Gastrectomy and colorectomy were performed in 51.4 and 48.6% of cases, respectively. Most procedures were performed laparoscopically (85.9%).

**Table 1:** Baseline characteristics and clinical outcomes of included patients (*N* = 642).

Variable	Value
Gender (female)	220 (34.3%)
Age (years)	60.34 ± 10.99
20–40	40 (6.2%)
41–60	262 (40.8%)
≥61	340 (53.0%)
BMI	24.10 ± 3.43
Underweight	16 (2.5%)
Normal weight	340 (53.0%)
Overweight	211 (32.8%)
Obesity	75 (11.7%)
Cancer stage	
I	167 (26.0%)
II	155 (24.1%)
III	273 (42.5%)
IV	47 (7.4%)
Surgery type	
Gastrectomy	330 (51.4%)
Colorectomy	312 (48.6%)
Operation approach	
Laparoscope	551 (85.8%)
Laparotomy	91 (14.2%)
NRS2002 (≥3)	474 (73.8%)
GLIM malnutrition (yes)	266 (41.4%)
Neoadjuvant (yes)	210 (32.7%)
Inflammation (yes)	480 (74.8%)
ALB (g/L)	40.42 ± 4.47
PALB (g/L)	0.19 (0.15–0.24)
CRP (mg/L)	20.40 (4.13–48.83)
Complications (yes)	84 (13.1%)
Infectious complications (yes)	48 (7.5%)

Data are presented as total number plus percentage (in parenthesis) among all participants [*n* (%)] or mean ± standard deviation or median (interquartile range), as appropriate. Only complications and infectious complications occured after postoperative day 2 and within 30 days after surgery were considered. Weight class was set according to the BMI (body mass index) definitions for adults by the Chinese guidelines (45). ALB, albumin; PALB, prealbumin; CRP, C-reactive protein.

GLIM-defined malnutrition was identified in 41.4% of patients, and 32.7% received neoadjuvant therapy. Overall, 84 patients (13.1%) developed postoperative complications occurring after POD 2 and within 30 days, including 48 cases (7.5%) of infectious complications. In fully adjusted logistic regression models examining baseline covariates, no individual variable reached statistical significance (all *p* > 0.05; Table 2).

**Table 2:** Fully adjusted logistic regression model for subsequent overall postoperative complications.

Variable	Adjusted OR	95% CI	*p*-value
Age (per year)	0.99	0.97–1.01	0.21
Gender (male vs. female)	1.17	0.71–1.96	0.55
BMI (per kg/m^2^)	0.99	0.93–1.06	0.85
Cancer stage III-IV vs. I-II	1.36	0.83–2.23	0.22
Surgery type (colorectomy vs. gastrectomy)	1.12	0.70–1.80	0.64
GLIM malnutrition (yes vs. no)	1.36	0.84–2.19	0.21
Neoadjuvant (yes vs. no)	0.93	0.55–1.57	0.80

Models adjust for age, BMI, gender, cancer stage, surgery type, GLIM-defined malnutrition, and preoperative neoadjuvant therapy. ALB, albumin; BMI, body mass index; CRP, C-reactive protein; GLIM, Global Leadership Initiative on Malnutrition; RCS, restricted cubic spline.

### 3.2 Postoperative energy intake: dose–response association and optimal range

**Figure 1:**
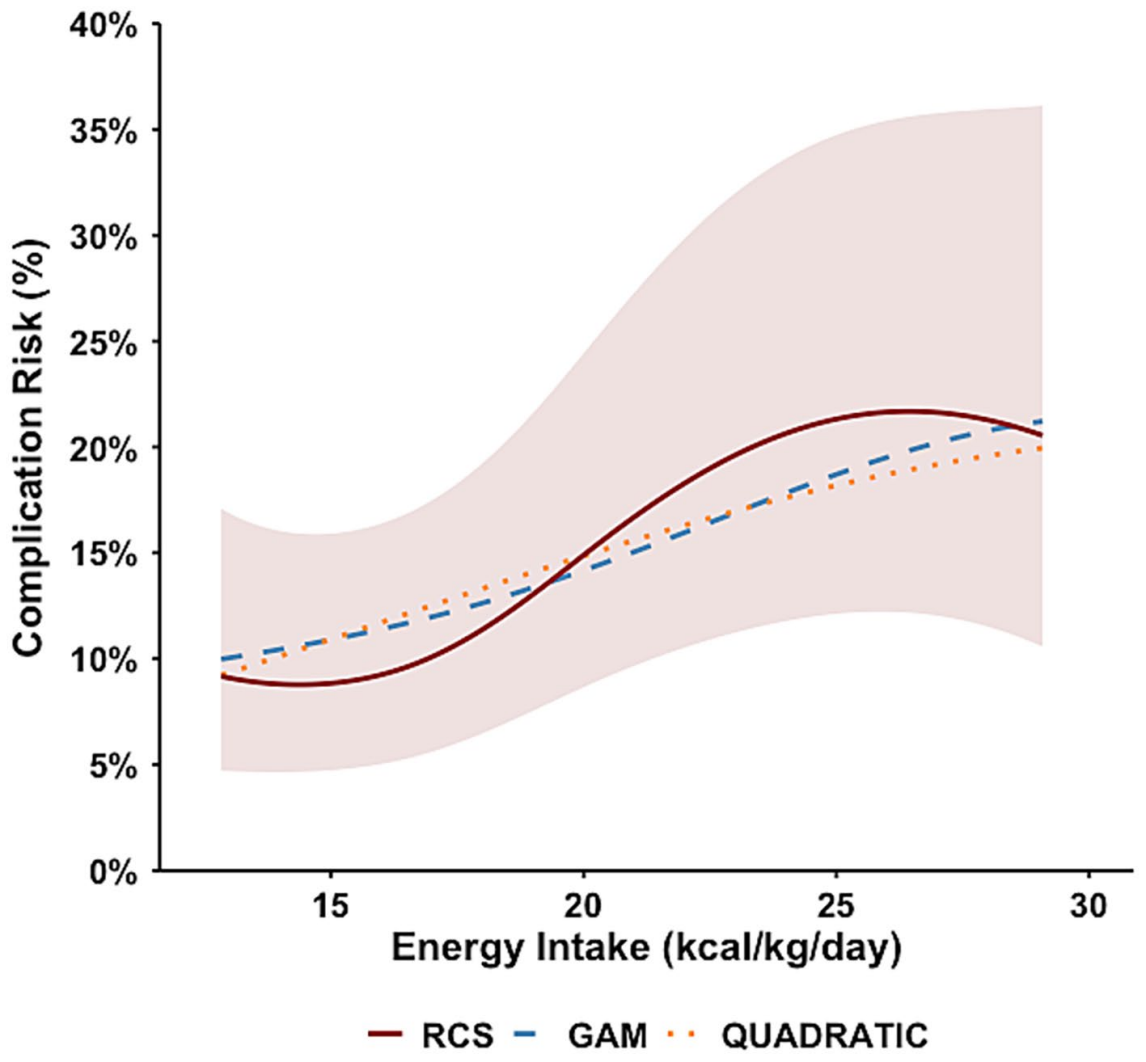
Energy intake and overall postoperative complications.

RCS analysis demonstrated a statistically significant nonlinear association between early postoperative energy intake and subsequent overall complications (likelihood ratio test for nonlinearity, *p* = 0.018) (Figure 1). The dose–response curve exhibited an oblique S-shaped pattern, characterized by decreasing predicted risk with increasing intake up to the nadir at 14.4 kcal/kg/day, followed by a gradual rise at higher intake levels. Using the predefined CI-overlap criterion, the optimal intake range was 12.8–20.1 kcal/kg/day.

GAM analysis suggested potential nonlinearity for energy intake, though this did not reach statistical significance (EDF = 2.09; Chi. sq. = 5.15; *p* = 0.098), with an estimated nadir of 12.8 kcal/kg/day (Supplementary Table S2). The quadratic model showed no significant association, with neither the linear (*β* = 0.149, *p* = 0.21) nor the quadratic term (*β* = −0.002, *p* = 0.38) reaching statistical significance (overall model *p* = 0.08) (Supplementary Table S3). The AUC for the RCS model including energy intake was 0.654 (Table 3). Bootstrap resampling with 1,000 iterations demonstrated stable estimation of the energy intake nadir and optimal range (Supplementary Table S4).

**Table 3:** Dose–response modeling results for subsequent overall postoperative complications.

Exposure	RCS Nadir	Optimal intake range	LR test *p*-value	AUC (RCS)
Energy (kcal/kg/day)	14.4	12.8–20.1	0.018	0.644
Protein (g/kg/day)	0.81	0.55–1.51	0.166	0.617

Optimal intake range is defined as exposure values where the lower bound of the 95% confidence interval of predicted risk did not exceed the minimum predicted risk. Bootstrap resampling (1,000 iterations) was used to estimate uncertainty.

### 3.3 Postoperative protein intake: dose–response association and optimal range

**Figure 2:**
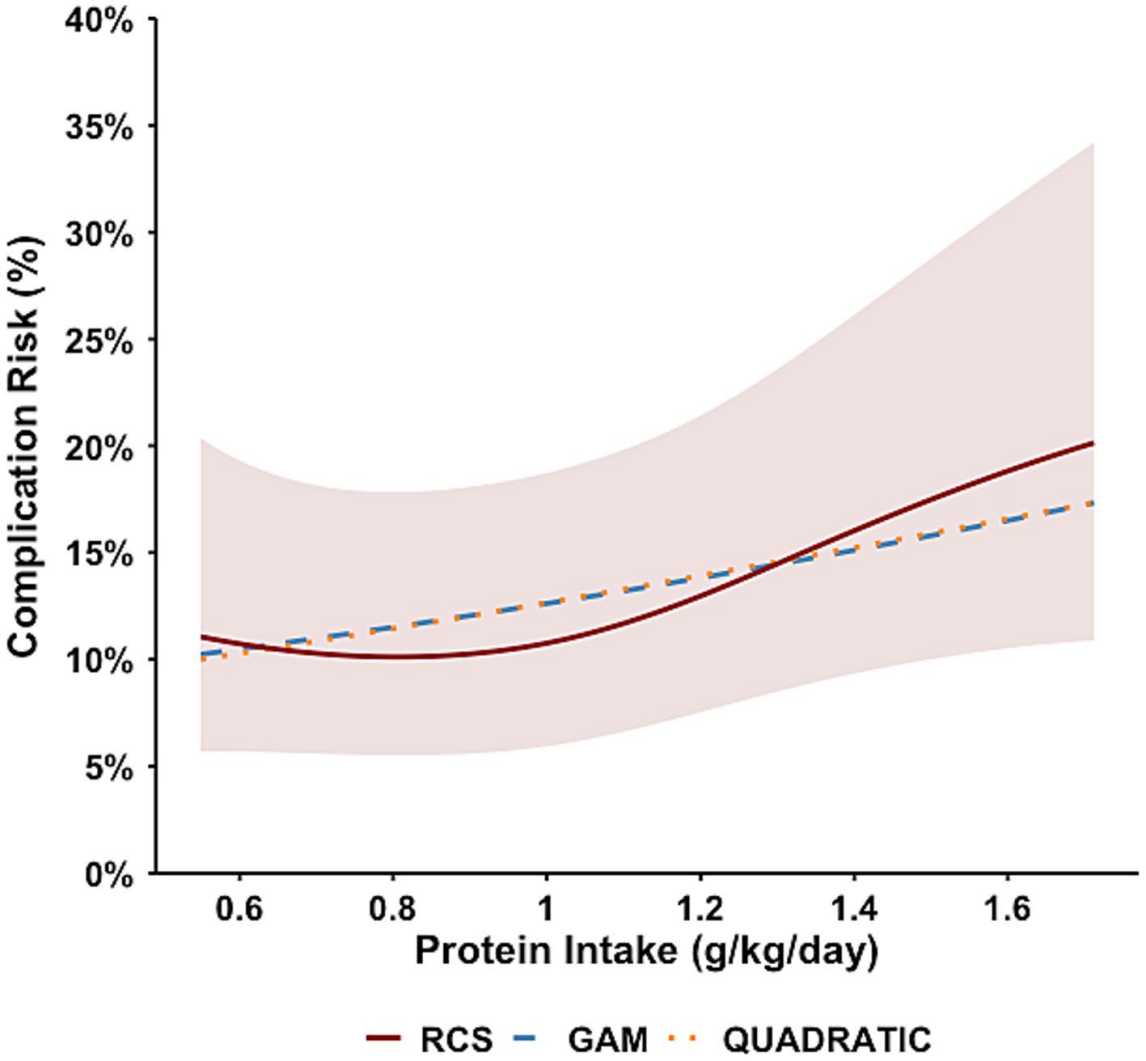
Protein intake and overall postoperative complications.

RCS analysis did not demonstrate statistically significant nonlinearity between early postoperative protein intake and subsequent overall complications (likelihood ratio test for nonlinearity, *p* = 0.166) (Figure 2). The estimated nadir occurred at 0.81 g/kg/day, with the estimated optimal intake range of 0.55–1.51 g/kg/day. However, given the absence of statistically significant nonlinearity and variability across modeling approaches, these findings should be interpreted cautiously.

GAM analysis showed no significant nonlinear association (EDF = 1.00; Chi.sq = 3.00; *p* = 0.084), with an estimated nadir of 0.55 g/kg/day (Supplementary Table S2). Similarly, quadratic regression revealed neither significant linear (*β* = 0.678, *p* = 0.42) nor quadratic (*β* = −0.058, *p* = 0.84) terms, and the overall model was not significant (*p* = 0.02) (Supplementary Table S3). The RCS protein model yielded an AUC of 0.617 (Table 3). Bootstrap validation (1000 iterations) indicated wider confidence intervals for protein-related nadir estimates compared with energy intake (Supplementary Table S4).

### 3.4 Secondary outcome: infectious complications

Among 48 patients (7.5%) with subsequent infectious complications, dose–response patterns were directionally consistent with those observed for subsequent overall complications, though with wider confidence intervals reflecting limited power (Supplementary Figures S1, S2; Supplementary Table S4). For energy intake, RCS analysis identified a nadir at 15.7 kcal/kg/day (optimal range: 12.8–29.1 kcal/kg/day), while nonlinearity was not statistically significant (*p* = 0.19). For protein intake, the estimated nadir was 0.84 g/kg/day (optimal range: 0.55–1.71 g/kg/day), also without significant nonlinearity (*p* = 0.20). Model discrimination was modest (AUC: energy 0.626, protein 0.605). In covariate-adjusted models, no individual covariate reached statistical significance (all *p* > 0.05; Supplementary Table S5).

### 3.5 Subgroup analyses

Prespecified subgroup analyses were performed to assess potential effect modification by GLIM-defined malnutrition status, surgery type, and preoperative neoadjuvant therapy (Supplementary Table S6). Effect estimates represent the change in odds of overall postoperative complications across the interquartile range of early postoperative energy intake.

Point estimates differed numerically between strata, with no statistically significant interactions detected between energy intake and any of the stratification factors examined (all *p*-for interaction >0.05).

### 3.6 Model performance and sensitivity analyses

Sensitivity analyses using alternative modeling strategies demonstrated broadly consistent nonlinear patterns for energy intake, with statistically significant nonlinearity observed in RCS and GAM models. For protein intake, results were consistently non-significant across modeling strategies.

All models were adjusted for age, BMI, sex, cancer stage, surgery type, GLIM-defined malnutrition, and neoadjuvant therapy. Multicollinearity diagnostics did not indicate significant collinearity among covariates. Model discrimination was modest, with AUC values ranging from 0.625 to 0.654.

## 4 Discussion

This prospective observational cohort study of 642 patients undergoing major GI cancer surgery provides a detailed evaluation of early postoperative nutritional dose–response relationships using flexible nonlinear modeling approaches. Several principal findings emerge. First, early postoperative energy intake demonstrated a statistically significant nonlinear association with overall complications, characterized by an asymmetric pattern with a nadir at approximately 14.4 kcal/kg/day and an estimated optimal range of 12.8–20.1 kcal/kg/day. Second, protein intake showed a weaker and less consistent association with complications, with no statistically significant evidence of nonlinearity and no clearly defined upper harm threshold within routinely observed clinical ranges. Third, secondary analyses of infectious complications showed broadly similar directional patterns but with lower discrimination and wider uncertainty intervals. Collectively, these findings refine current understanding of early postoperative nutritional exposure in surgical oncology and highlight the potential importance of moderation during the immediate postoperative period.

The most robust finding of this study is the nonlinear and asymmetric relationship between early postoperative energy intake and complications. The nadir at 14.4 kcal/kg/day (optimal range: 12.8–20.1) lies below conventional weight-based energy estimates of approximately 25–30 kcal/kg/day commonly referenced in ESPEN and ASPEN guidance when indirect calorimetry is unavailable (8, 9). These guideline-derived estimates are largely extrapolated from critical illness literature and predictive equations, rather than from dose–response analyses specific to elective GI cancer surgery. Our results therefore suggest that energy requirements during the immediate postoperative period may differ from generalized acute illness recommendations, particularly during the early stress phase.

Several physiological mechanisms may plausibly underlie this nonlinear association. Excessive caloric provision during the immediate postoperative phase may exacerbate metabolic stress responses through hyperglycemia, insulin resistance, increased hepatic lipogenesis, and mitochondrial substrate overload, particularly in patients with malignancy-associated alterations in intermediary metabolism (9, 25). Surgical stress induces a transient state of reduced metabolic flexibility and impaired oxidative capacity, potentially limiting tolerance to aggressive caloric delivery in the early recovery window (12, 26). In our cohort, 41.4% of patients met GLIM criteria for malnutrition, reflecting substantial baseline metabolic vulnerability. Cancer-associated inflammation and cachexia are characterized by altered energy partitioning, systemic cytokine activation, and reduced anabolic responsiveness, all of which may reduce the efficiency with which exogenous energy is utilized (16, 27). In such a context, overfeeding may impose additional metabolic burden without proportionate anabolic benefit.

Conversely, inadequate energy provision during this acute catabolic phase may compromise immune function, collagen synthesis and tissue repair, increasing susceptibility to postoperative complications (28, 29). Our findings align with emerging evidence questioning aggressive early caloric targets. Randomized trials in critically ill adults have demonstrated no benefit, and potential harm, of full early feeding compared with permissive hypocaloric strategies (11, 30, 31). Although extrapolation from critical care to elective surgical populations requires caution, these findings collectively support a more restrained approach to early postoperative energy provision.

A meta-analysis of perioperative nutritional interventions in gastric cancer patients confirmed that while nutritional support can improve outcomes, substantial heterogeneity across studies highlights the need for precise, quantitative targets for optimal energy delivery (32). Comparatively limited observational and interventional evidence in surgical cohorts suggests that moderate early caloric delivery (approximately 15–20 kcal/kg/day) may be associated with fewer complications compared with higher caloric targets (33, 34). Our modeling results are consistent with this moderate intake range. Notably, convergence across RCS, GAM, and quadratic modeling approaches, together with a statistically significant likelihood ratio test for nonlinearity (*p* = 0.018) in the RCS model, strengthens confidence that the observed association is robust to model specification. Bootstrap validation (1,000 iterations) further supports the stability of the estimated nadir.

In contrast, protein intake demonstrated a more modest and statistically less robust relationship with postoperative complications. Restricted cubic spline modeling suggested a shallow nadir at approximately 0.81 g/kg/day; however, likelihood ratio testing did not support significant nonlinearity (*p* = 0.166). GAM and quadratic models suggested predominantly monotonic or weakly nonlinear patterns across the exposure distribution, with the lowest predicted risk observed at the lowest observed intake. Across all modeling approaches, predicted risk remained relatively similar within the approximate range of 0.55–1.51 g/kg/day, and no consistent upper harm threshold was observed within routine practice. These findings therefore warrant cautious interpretation.

These results are biologically plausible. Surgical stress and malignancy-associated catabolism promote accelerated proteolysis and negative nitrogen balance, theoretically increasing protein requirements compared with healthy states (35, 36). While improved nitrogen balance and preservation of lean mass have been observed with higher protein delivery in hospitalized populations, improvements in surrogate endpoints do not consistently translate into reduced postoperative complications (37, 38). During the early postoperative period, metabolic stress, systemic inflammation, and insulin resistance may blunt anabolic signaling pathways, limiting the incremental benefit of escalating protein provision beyond moderate levels. Thus, although avoidance of very low protein intake appears reasonable, aggressive early escalation may not uniformly reduce short-term complication risk.

Recent randomized evidence further underscores the uncertainty surrounding high protein targets. The PRECISe trial reported worse health-related quality of life with high protein provision (2.0 g/kg/day) compared with standard provision (1.3 g/kg/day) in critically ill adults, with Bayesian re-analysis suggesting potential harm (20, 21). While these exposures exceed those typically achieved in early postoperative surgical care and involve prolonged ICU settings, they highlight that greater protein provision is not necessarily beneficial across all clinical contexts. In our cohort, protein intake generally remained below 1.8 g/kg/day, and no clear upper harm threshold was identified. The weaker and less consistent association for protein intake, compared with energy, suggests that the weaker and less consistent association for protein intake, compared with energy, suggests that, within the observed ranges, energy intake demonstrated a more consistent association with outcomes than protein intake.

Methodologically, a key strength of this study lies in the application of complementary nonlinear modeling strategies, namely RCS, GAM, and quadratic regression, to characterize continuous exposure–response relationships. This multipronged approach reduces reliance on arbitrary categorization and mitigates risks of model misspecification inherent in strictly linear analyses (39). Compared with GAM, RCS provides a parametric spline framework within conventional regression modeling, allowing direct likelihood ratio testing, easier reproducibility, and clearer estimation of clinically interpretable features such as nadir points and exposure ranges. The absence of statistical significance in quadratic regression likely reflects its parametric constraint to a symmetric U-shaped curve, which may not adequately capture the asymmetric dose–response pattern observed in spline-based modeling. Thus, RCS, which allows greater flexibility, was prioritized for inference. By quantifying nadirs, estimating confidence intervals through bootstrap resampling, and defining optimal intake ranges using a transparent CI-overlap criterion, the analysis advances beyond traditional threshold-based interpretations (23, 24). Greater convergence across models for energy intake, compared with protein, supports the robustness of the observed association.

Nutritional studies are vulnerable to immortal time bias when intake is measured over variable durations extending until event occurrence (40). The present analysis restricted outcomes to complications occurring after the exposure window (POD 1-2), thereby strengthening temporal ordering and reducing likelihood of reverse causation. Comprehensive adjustment for age, BMI, gender, cancer stage, surgery type, GLIM-defined malnutrition, and preoperative neoadjuvant therapy strengthens confounder control, though residual confounding cannot be excluded.

Several practical implications emerge. First, moderate early energy provision, approximately 12–20 kcal/kg/day during POD 1-2, was associated with the lowest predicted complication risk in this cohort. This range is lower than conventional guideline targets and may better reflect the metabolic constraints of the immediate postoperative stress phase. Second, protein delivery within approximately 0.5–1.5 g/kg/day appeared compatible with relatively low complication risk, although no sharply defined optimal threshold was identified. These findings suggest that cautious titration, rather than aggressive early escalation, may be appropriate in the immediate postoperative setting.

Importantly, nutritional prescriptions should remain individualized. Baseline malnutrition status, inflammatory burden, surgical magnitude, cancer stage, and metabolic comorbidities may influence tolerance to early nutritional exposure. Integration of moderate early energy strategies into ERAS pathways may warrant consideration, particularly in light of ongoing efforts to refine perioperative metabolic care (41). However, translation into clinical guidelines requires confirmation in randomized trials.

The precision of covariate effect estimates differed between overall and infectious complication models, reflecting the relationship between event count and statistical stability. With 84 overall events, estimates were reasonably precise, whereas the smaller number of infectious events (*n* = 48) resulted in wider confidence intervals and less stable estimates. Accordingly, secondary outcome analyses are exploratory, and primary inferences should rely on overall complications.

In pre-specified subgroup analyses, the association between early postoperative energy intake and overall complications appeared directionally similar across strata defined by GLIM-defined malnutrition, surgery type, and preoperative neoadjuvant therapy. Although point estimates were numerically higher in certain subgroups, formal interaction testing did not demonstrate statistically significant effect modification. These findings suggest that the observed dose–response relationship was broadly consistent across clinically relevant patient subsets; however, the study was not powered specifically to detect interaction effects, and subgroup findings should be considered exploratory and require validation in larger cohorts.

This study has several strengths, including its prospective design, relatively large and clinically homogeneous cohort of GI cancer surgery patients, detailed early postoperative intake measurement, and rigorous application of nonlinear modeling with bootstrap validation. The fixed exposure window reduces immortal time bias, and adjustment for GLIM-defined malnutrition strengthens confounder control (42).

*Limitations merit consideration*: As an observational study, causal inference cannot be established despite multivariable adjustment. Residual confounding cannot be excluded, particularly confounding by indication, whereby patients with greater clinical severity may be more likely to tolerate or receive lower nutritional intake and also have a higher baseline risk of complications. In addition, detailed perioperative variables such as operative duration, intraoperative hemodynamics, glycemic control, and early postoperative clinical severity were not available for adjustment and may have influenced both nutritional exposure and outcomes. Nutritional exposure was derived from recorded intake rather than direct measurement of energy expenditure, and indirect calorimetry was not performed; therefore, intake may not reflect individualized metabolic requirements. The fixed exposure window (POD 1-2) was used to minimize reverse causation, but this approach does not capture cumulative nutritional intake during the broader postoperative recovery period. Although we used flexible modeling and bootstrap validation, the identified nadir and optimal ranges are model-derived and may be influenced by the underlying exposure distribution and modeling assumptions. The single-center design may limit generalizability, although standardized perioperative care in a high-volume cancer center enhances internal consistency. The high proportion of laparoscopic procedures in this cohort may further limit generalizability to settings with different surgical practices, particularly where open surgery is more commonly performed. Additionally, outcomes were limited to short-term postoperative complications; longer-term endpoints such as functional recovery, body composition changes, oncologic outcomes, and survival were not evaluated.

Future investigations should validate these dose–response relationships in multicenter surgical cohorts and across diverse cancer types and procedures. Randomized trials comparing moderate versus conventional early energy targets, while holding protein delivery constant, are needed to determine causality and clinical impact. Adaptive trial designs incorporating metabolic biomarkers, indirect calorimetry, insulin sensitivity assessment, inflammatory profiling, and longitudinal body composition analysis may further clarify mechanisms and refine individualized nutritional strategies (43, 44). Additionally, adequately powered subgroup analyses are required to determine whether optimal intake ranges differ according to malnutrition status, cancer stage, inflammatory burden, or surgical approach.

## 5 Conclusion

In this prospective cohort of patients undergoing gastrointestinal cancer surgery, early postoperative energy intake demonstrated a statistically significant nonlinear association with subsequent postoperative complications, with the lowest predicted risk observed at moderate levels of early caloric provision. The identified optimal range was lower than some traditional weight-based perioperative estimates. In contrast, protein intake showed no statistically significant evidence of nonlinearity and exhibited less consistent dose–response patterns within commonly observed clinical ranges.

These findings suggest that moderate early caloric provision, rather than aggressive escalation, may be associated with lower complication risk following gastrointestinal cancer surgery. For protein intake, the results should be interpreted cautiously given the absence of statistically significant associations and require further validation. Overall, the present study provides hypothesis-generating evidence rather than practice-changing recommendations. Prospective validation, including randomized evaluation of moderate versus conventional early energy targets, is warranted to determine clinical effectiveness and inform future perioperative nutritional guidelines.

## Data Availability

The original contributions presented in the study are included in the article/Supplementary material, further inquiries can be directed to the corresponding authors.
