# Supplementary material for: Dose–response relationships between early postoperative nutrition and subsequent complications in gastrointestinal cancer: optimal intake ranges for energy and protein
Source: Front Nutr. 2026 May 13;13:1826047. doi: 10.3389/fnut.2026.1826047 (PMC13212077; doi:10.3389/fnut.2026.1826047)
Supplement: Supplementary file 1 [file Table_1.docx]

Supplementary Material

# Supplementary Figures and Tables

## **TABLE S1.** Detailed Inclusion, Exclusion, and Withdrawal Criteria.

| **Category** | **Criteria** |
| --- | --- |
| Inclusion Criteria |  |
| Age | 18–90 years |
| Diagnosis | Histologically confirmed stage I–IV gastric or colorectal malignancy |
| Surgery type | First elective gastrointestinal resection (gastrectomy or partial colectomy) |
| Performance status | ECOG performance status 0–2 |
| Hospitalization | Planned postoperative hospital stay ≥5 days |
| Nutritional assessment | Ability to complete perioperative nutritional assessments and documented intake during POD 1–2 |
| Follow-up | Availability of 30-day postoperative follow-up data |
| Exclusion Criteria |  |
| Tumor type | Carcinoma in situ, stromal tumors, benign disease, or non-gastrointestinal malignancies |
| Surgery characteristics | Emergency surgery, multi-organ resection, or prior major abdominal surgery |
| Admission type | Admission solely for neoadjuvant therapy or non-surgical medical management |
| Organ dysfunction | Severe preoperative cardiac, renal, hepatic, neurological, or other significant organ dysfunction |
| Clinical conditions | Presence of ascites, cachexia, edema, confusion, severe diarrhea, gut dysbiosis, or preoperative bleeding/obstruction unrelated to the surgical site |
| Nutritional support | Inadequate perioperative nutritional support (<5 days of combined enteral and/or parenteral nutrition) |
| Logistical factors | Preoperative hospitalization <24 hours, postoperative stay <5 days, or scheduling constraints interfering with study procedures |
| Data completeness | Missing primary outcome data or inability to complete required study procedures |
| Other | Patient refusal, investigator discretion, or concurrent participation in interventional nutrition trials |
| Withdrawal Criteria (Dropout During Study) |  |
| ICU transfer | Unplanned transfer to the intensive care unit during hospitalization |
| Discontinuation | Patient-initiated withdrawal or loss to follow-up |
| Safety/protocol | Investigator-determined withdrawal due to safety concerns, emerging exclusion criteria, or protocol violations |

## **TABLE S2.** Generalized additive model (GAM) analysis of dose–response relationships.

| **Exposure** | **Nadir** | **EDF** | **Ref.df** | **Chi.sq** | ***p* value** |
| --- | --- | --- | --- | --- | --- |
| Energy intake (kcal/kg/day) | 12.8 | 2.09 | 2.53 | 5.146 | 0.098 |
| Protein intake (g/kg/day) | 0.55 | 1.00 | 1.00 | 2.995 | 0.084 |

Note: Models adjust for age, BMI, gender, cancer stage, surgery type, GLIM-defined malnutrition, and preoperative neoadjuvant therapy. EDF, estimated degrees of freedom. EDF > 1 suggests potential nonlinearity, with statistical significance determined by the p value.

## **TABLE S3.** Quadratic regression analysis of nutritional intake and postoperative complications.

| **Exposure** | **Nadir** | **Linear term β (SE)** | **p value** | **Quadratic term β (SE)** | ***p* value** | **Overall model *p* value** |
| --- | --- | --- | --- | --- | --- | --- |
| Energy intake (kcal/kg/day) | 12.8 | 0.149 (0.118) | 0.21 | -0.002 (0.0026) | 0.38 | 0.08 |
| Protein intake (g/kg/day) | 0.55 | 0.678 (0.840) | 0.42 | -0.058 (0.2949) | 0.84 | 0.22 |

Note: Models adjust for age, BMI, gender, cancer stage, surgery type, GLIM-defined malnutrition, and preoperative neoadjuvant therapy.

## **TABLE S4.** Dose–response modeling results for subsequent infectious postoperative complications.

| **Exposure** | **RCS Nadir** | **Optimal intake range** | **LR Test *p* value** | **AUC (RCS)** |
| --- | --- | --- | --- | --- |
| Energy intake (kcal/kg/day) | 15.7 | 12.8–29.1 | 0.19 | 0.626 |
| Protein intake (g/kg/day) | 0.84 | 0.55–1.71 | 0.17 | 0.605 |

Note: Optimal intake range is defined as exposure values where the lower bound of the 95% confidence interval of predicted risk did not exceed the minimum predicted risk. Bootstrap resampling (1000 iterations) was used to estimate uncertainty.

## **TABLE S5.** Fully adjusted logistic regression model for subsequent infectious complications.

| **Variable** | **Adjusted OR** | **95% CI** | ***p* value** |
| --- | --- | --- | --- |
| Age (per year) | 1.00 | 0.97–1.03 | 0.95 |
| Gender (Male vs Female) | 1.63 | 0.83–3.38 | 0.17 |
| BMI (per kg/m²) | 1.00 | 0.91–1.08 | 0.93 |
| Cancer stage III–IV vs I–II | 0.94 | 0.50–1.78 | 0.86 |
| Surgery type (Colorectomy vs Gastrectomy) | 1.19 | 0.65–2.20 | 0.57 |
| GLIM malnutrition (Yes vs No) | 1.14 | 0.61–2.11 | 0.68 |
| Neoadjuvant (Yes vs No) | 1.42 | 0.73–2.72 | 0.29 |

Note: Models adjust for all covariates included in the primary analysis. ALB, albumin; BMI, body mass index; CRP, C-reactive protein; GLIM, Global Leadership Initiative on Malnutrition; RCS, restricted cubic spline.

**TABLE S6.** Stratified analyses of the association between early postoperative energy intake and subsequent overall complications.

| **Stratification Factor** | **Subgroup** | **n** | **Effect Estimate** | **95% CI** | ***p* value** | ***p* for interaction** |
| --- | --- | --- | --- | --- | --- | --- |
| GLIM-defined malnutrition |  |  |  |  |  | 0.45 |
|  | No | 376 | 1.17 | 0.82–1.66 | 0.39 |  |
|  | Yes | 266 | 1.48 | 1.00–2.20 | 0.05 |  |
| Surgery type |  |  |  |  |  | 0.91 |
|  | Gastrectomy | 330 | 1.32 | 0.89–1.95 | 0.17 |  |
|  | Colorectomy | 312 | 1.28 | 0.92–1.80 | 0.15 |  |
| Neoadjuvant therapy |  |  |  |  |  | 0.23 |
|  | Yes | 210 | 1.65 | 1.03–2.64 | 0.04 |  |
|  | No | 432 | 1.21 | 0.88–1.66 | 0.24 |  |

Note: Effect estimate reflects the change in odds of overall postoperative complications across the interquartile range of early postoperative energy intake. Models were adjusted for age, BMI, gender, cancer stage, surgery type (except where stratified), GLIM-defined malnutrition (except where stratified), and preoperative neoadjuvant therapy (except where stratified). Sample sizes (n) reflect number of patients in each subgroup from the total cohort of 642.


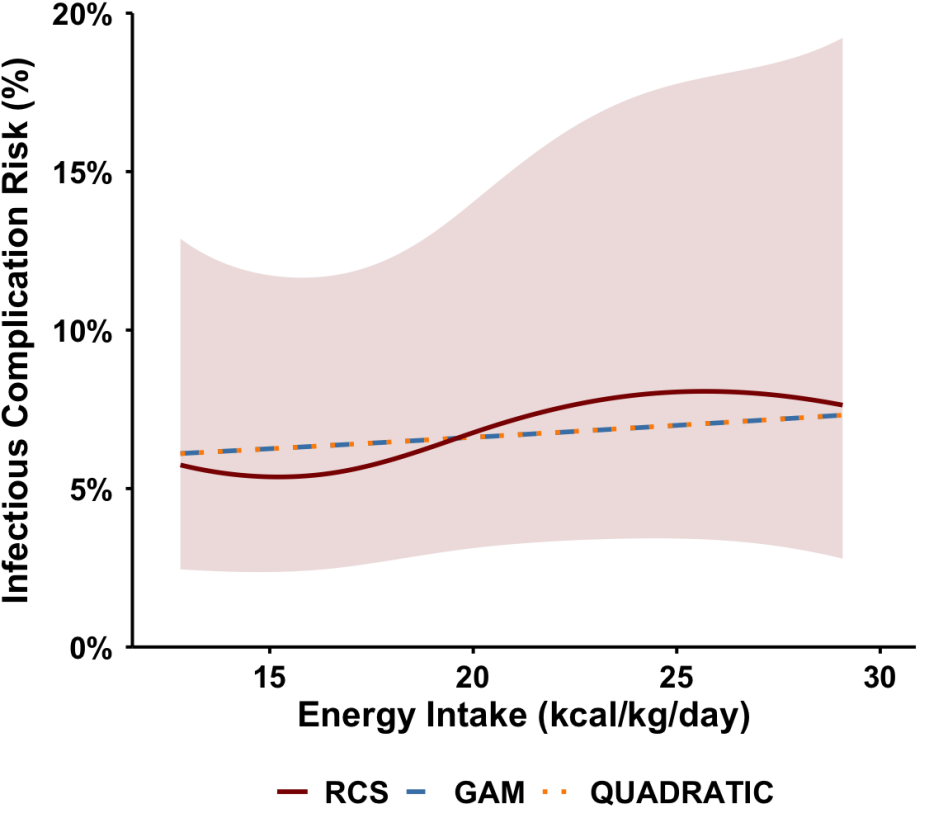


**FIGURE S1.** Energy Intake and Infectious Postoperative Complications.


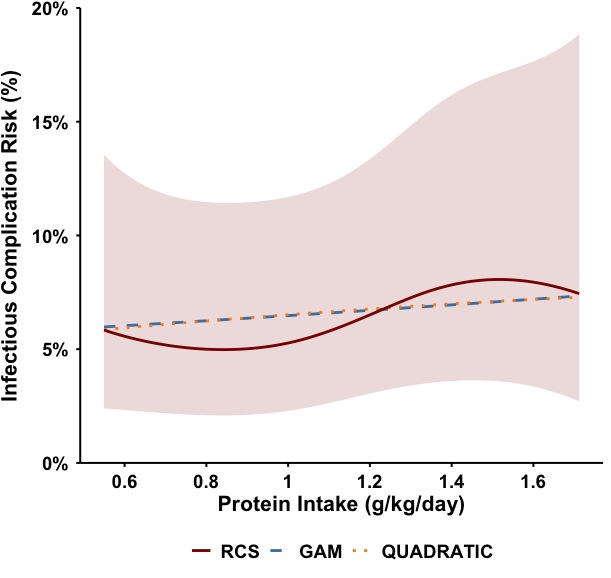


**FIGURE S2.** Protein Intake and Infectious Postoperative Complication.
